# Single-mode characteristic of a supermode microcavity Raman laser

**DOI:** 10.1073/pnas.2101605118

**Published:** 2021-05-25

**Authors:** Pei-Ji Zhang, Qing-Xin Ji, Qi-Tao Cao, Heming Wang, Wenjing Liu, Qihuang Gong, Yun-Feng Xiao

**Affiliations:** ^a^State Key Laboratory for Mesoscopic Physics, School of Physics, Peking University, 100871 Beijing, China;; ^b^Frontiers Science Center for Nano-optoelectronics, Peking University, 100871 Beijing, China;; ^c^Collaborative Innovation Center of Extreme Optics, Shanxi University, Taiyuan 030006, China;; ^d^Yangtze Delta Institute of Optoelectronics, Peking University, Nantong 226010, China

**Keywords:** optical microcavity, stimulated Raman scattering, near-degenerate modes, microlasers

## Abstract

Microlasers in near-degenerate supermodes lay the cornerstone for studies of non-Hermitian physics, novel light sources, and advanced sensors. Recent experiments of the stimulated scattering in supermode microcavities reported beating phenomena, interpreted as dual-mode lasing, which, however, contradicts their single-mode nature due to the clamped pump field. Here, we investigate the supermode Raman laser in a whispering-gallery microcavity and demonstrate experimentally its single-mode lasing behavior with a side-mode suppression ratio (SMSR) up to 37 dB, despite the emergence of near-degenerate supermodes by the backscattering between counterpropagating waves. Moreover, the beating signal is recognized as the transient interference during the switching process between the two supermode lasers. Self-injection is exploited to manipulate the lasing supermodes, where the SMSR is further improved by 15 dB and the laser linewidth is below 100 Hz.

## Significance

Microlasers in near-degenerate supermodes play a pivotal role for studies of non-Hermitian physics, novel light sources, and advanced sensors. However, the widely observed beating phenomena and the single-mode nature of the stimulated scattering have been generating the lasing characteristic dilemma. In this article, we experimentally elucidate the lasing spectral paradox by investigating the lasing dynamics of supermode Raman microlasers. The single-mode lasing behavior is confirmed, and the beating signal has been recognized as the transient interference during the lasing switching between the supermodes with the help of the self-injection technique. This work provides an insightful guidance for microlaser-based precision measurements and paves the way to reconfigurable light sources.

Microlasers in near-degenerate supermodes have drawn much attention in the past decades, promoting various advances such as spontaneous symmetry breaking (1, 2), exceptional points (3–6), precise measurement (7–11), and novel light sources (12–16). Single microcavities supporting high-Q whispering-gallery modes (WGMs) (17–19) are found as a natural platform for studying supermodes, which are formed by the coupling between degenerate counterpropagating waves (20–22). So far, supermode lasers in WGM microcavities have been demonstrated with not only intrinsic gain materials (3, 7, 12–14), but also nonlinear optical effects, e.g., stimulated scattering (2, 8, 9, 11). In particular, the latter is advantageous for recording low linewidths (23–25), as well as neither a requirement of specific gain materials nor a limitation in operation frequencies (26–30). Given that the energy splitting of supermodes is sensitive to the external perturbation (31, 32), the stimulated scattering, such as Raman or Brillouin lasers in supermode microcavities, has also shown unique merit for nanoparticle detection (8, 9), an exceptional-point-enhanced optical gyroscope (11, 33), and an Earth rotation reader (34), featuring a beat note corresponding to the splitting.

Different from the conventional inversion lasers based on electronic transitions, stimulated scattering involving only bosonic modes is inherently immune from the spatial hole burning and holds a homogeneous gain (35–38). Hence, energy in the pump field should be clamped at a fixed value once the lasing threshold is reached (24, 39, 40), leading to a single-mode lasing (2, 6, 23). However, beat notes are widely observed in supermode microlaser output during pump scanning, so that these lasers are generally regarded as dual-mode lasers (8, 9, 41). In this work, we elucidate this lasing spectral paradox by investigating the dynamics of a supermode Raman laser in an ultrahigh-*Q* microcavity. Experimentally, the clamping effect on the pump field is demonstrated, confirming the single-mode nature of the supermode laser. When a tiny reflection is introduced and provides self-injection of the Raman laser, beating phenomena are observed and recognized as the transient interference during the lasing switching between the supermodes.

**Fig. 1.**
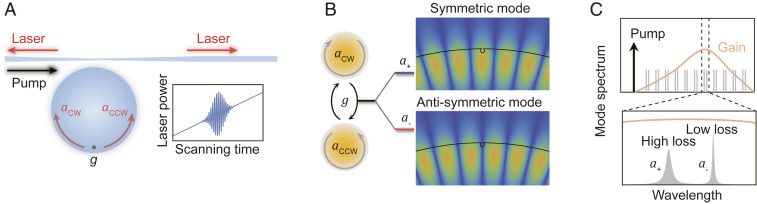
Schematic of supermode laser in a microcavity. (*A*) Counterpropagating waves in a WGM cavity are coupled by a defect with strength g, forming a pair of supermodes. (*Inset*) Schematic of the beating phenomenon during pump scanning. (*B*) Formation of the symmetric $a_{+}$ and antisymmetric $a_{-}$ supermodes. (*Right*) Field distribution of the two supermodes. (*C*, *Upper*) The cavity modes (gray lines) in the frequency domain and the optical gain (orange curve) from the pump (black line). (*C*, *Lower*) The zoomed-in spectrum of the black dashed box in *C*, *Upper*.

The Raman microlaser is generated by optically pumping a WGM microcavity and propagates in both the clockwise (CW) and counterclockwise (CCW) directions (Fig. 1*A*) (2, 26). The intracavity counterpropagating waves are coupled by a scatterer at the surface (8, 9, 21, 42), which forms a pair of standing-wave supermodes, the symmetric $a_{+}$ and antisymmetric $a_{-}$, defined by the relative position between the mode distribution and the scatterer (Fig. 1*B*). If the scatterer is a dielectric particle at the surface (or a vacancy-like defect), the symmetric supermode has a lower (or higher) resonance frequency and larger decay rate (31). In addition, here the Raman gains in the symmetric mode are slightly smaller due to the different stimulated Raman scattering rates in the counterpropagating directions (43, 44) (*Materials and Methods*). According to the mode competition theory, only the mode with the smaller loss can reach the lasing condition (45), accompanied with the clamped pump field. Despite the previous observation of the clamping effect with the assistance of parametric oscillation (46) or cascaded stimulated scattering (24, 47), the clamped input pump characterizing the single-mode lasing has not been directly demonstrated in the supermode microlasers so far, while beating phenomena (Fig. 1*A*) were widely observed and generally interpreted as a dual-mode signature.

**Fig. 2.**
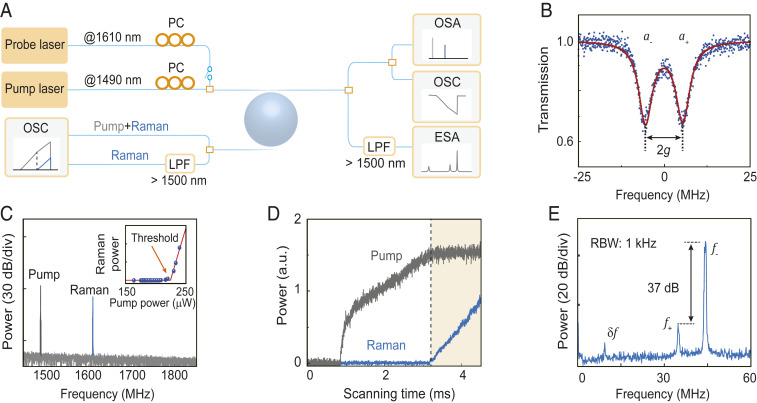
Experimental characterizations of the microcavity Raman laser. (*A*) Experimental setup. PC, polarization controller; OSA, optical spectrum analyzer; OSC, oscilloscope; ESA, electrical spectrum analyzer; LPF, long-pass filter. (*B*) Transmission spectrum of the supermodes and the theoretical fitting. (*C*) Optical spectrum of the Raman laser. (*Inset*) Threshold curve of the Raman laser. (*D*) Experimental observation of the clamping effect on the pump field. (*E*) Frequency spectrum of the combined probe light and Raman emission, in which the wavelength of the pump beam is unchanged.

To investigate the supermode Raman lasing, a silica microsphere cavity with intrinsic Q factor over $4\times 10^{7}$ is applied, as illustrated in Fig. 2*A*. A tapered fiber is evanescently coupled to the cavity, and the transmission of the Stokes supermodes is measured, as shown in Fig. 2*B* (*Materials and Methods*). The doublet indicates a coupling strength of g=5.49±0.01 MHz between the counterpropagating waves. The decay rates of the symmetric and antisymmetric modes are fitted to be $\kappa_{+0}/2\pi =4.05\pm 0.05$ MHz and $\kappa_{-0}/2\pi =3.93\pm 0.05$ MHz, respectively. The fact that the mode at lower frequency features smaller loss indicates that the scatterer is a vacancy-like defect. By tuning the pump laser (∼1,490 nm) into the resonance of the cavity mode, the first-order Raman laser is observed as a single line at 1,610 nm (Fig. 2*C*) with the threshold of 213 μW, where the cascaded Raman laser is absent (39, 48). Note that multiline Raman lasers were also reported previously with the presence of Kerr parametric gain (49, 50) or cascaded scattering gain (51), which are avoided in the present work by carefully controlling the pump power as well as the coupling condition. The clamping of the pump field is then examined by monitoring the intracavity pump power via an add–drop coupling scheme (22, 52). As the pump laser with a constant power scans from the blue-detuned region toward the resonance, the intracavity pump power increases and reaches the threshold, after which the power of the Raman laser grows monotonically. Simultaneously, the intracavity pump power is clamped at a constant value (Fig. 2*D*), indicating that the gain, matching the loss of the higher-*Q* supermode, remains unchanged. Hence, the loss of the other supermode cannot be compensated, and the laser operates in the single-mode regime.

Quantitatively, a probe laser with the frequency slightly higher than the supermode is introduced to interfere with the output laser, and the frequency spectrum is presented in Fig. 2*E*. The peak with the center frequency of $f_{+}$ ($f_{-}$) is attributed to the interference between the probe laser and the signal from the symmetric (antisymmetric) supermode, while the tiny peak located at δf=12.5 MHz corresponds to the interference between the two supermodes. Here, the beating frequency δf, equal to $f_{-}-f_{+}$, is slightly larger than the splitting of the passive Raman supermodes due to the Kerr effect-induced mode shift (*Materials and Methods*) (22). The intensity of the $f_{-}$ peak is much higher than that of the $f_{+}$ peak, manifesting a large side-mode suppression ratio (SMSR) over three orders of magnitude. The large SMSR, as a widely adopted criterion, hence indicates that the supermode Raman laser operates as a single-mode laser (12, 53). Additionally, considering the observed clamping effect, the weak signal in the symmetric mode is inferred as the amplified spontaneous emission, with a narrowed linewidth due to Raman gain compensation.

**Fig. 3.**
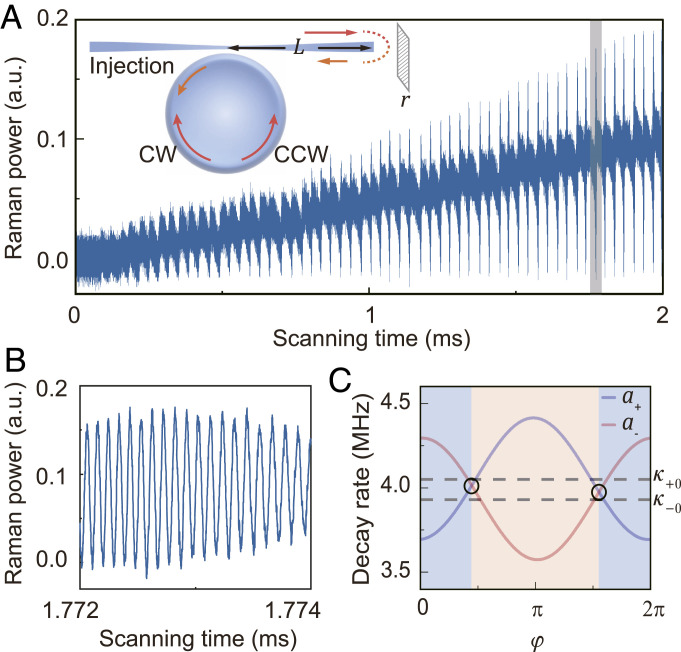
Beat notes of the supermode Raman laser with self-injection. (*A*) Real-time output of the Raman laser. (*Inset*) Schematic of the self-injected laser. (*B*) Zoom-in of the gray area in *A*, where a typical beat note is observed. (*C*) Theoretical dissipation of the two supermodes versus injection phase shift φ. Blue (orange) shading: Symmetric (antisymmetric) mode lasing regime. The black circles denote the lasing mode switching point at the particular injection phase.

Despite the presence of δf, temporal oscillations in the laser output cannot be observed due to the large SMSR (Fig. 2*E*), which contradicts the strong beat notes reported previously (8, 9). To reveal the underlying physics, we study the lasing dynamics dependent on the loss difference of the two supermodes. Thus, the self-injection technique is introduced to modulate the mode loss, which is widely utilized to regulate the intracavity laser field through the interference effect (54–56). Experimentally, a flat end face of the output fiber, serving as a reflector with a reflectance of only 0.033, reinjects partial output laser into the cavity (Fig. 3*A*, *Inset*). During the pump wavelength scanning, the beat note appears periodically, featured as the spike-like envelopes consisting of fast oscillations with a center frequency of 9.7 MHz (Fig. 3*B*). This beating frequency slightly deviates from the passive splitting as a result of the Kerr frequency shift (*Materials and Methods*).

Considering the interaction of CW-CCW lasers with self-injection, we write the system Hamiltonian H under the traveling-wave basis $\left(a_{cw},\;a_{ccw}\right)$ to investigate the lasing dynamics,

$$H=\left(\begin{array}{cc} \omega +i\frac{\kappa }{2} & g+i\frac{\gamma }{2}\\ g+i\frac{\gamma }{2}-i\tilde{r}\kappa_{in} & \omega +i\frac{\kappa }{2} \end{array}\right).$$ [1]

Here ω and κ are the unperturbed resonant frequency and mode loss, respectively, and γ is the dissipative coupling strength. $\kappa_{\text{in}}$ represents the external coupling rate, and $\tilde{r}$ denotes the complex reflectivity accounting for the optical-path–related phase and the reflectance at the end facet. In a realistic system, the coupling strength is large enough, i.e., $\left|g|\gg |\gamma /2|,\;|\tilde{r}\kappa_{in}\right|$, and thus the decay rates of the supermodes can be approximately obtained by eigenvalue analysis (*Materials and Methods*), as

$$\kappa_{\pm }=\kappa \pm \gamma \mp |\tilde{r}|\kappa_{in}\cos\left(\varphi \pm 2ngL/c\right),$$ [2]

with the injection phase φ=2nωL/c related to the material refractive index n and fiber length L between the coupling point and the reflector. The intrinsic loss difference between the two supermodes can be compensated and reversed by tuning the injection phase, the reflectivity, and the coupling rate, resulting in the switching of the supermode laser.

The modulation of the lasing status results from the interference between the reinjected wave and the original laser fields. The reinjected wave $a_{in,ccw}$ from the reflected CW output laser will contribute coherently to the intracavity CCW field. For the symmetric and antisymmetric supermodes, the CW and CCW fields are in phase and out of phase, respectively. Hence $a_{in,ccw}$ constructively interfering with the CCW field of one mode will destructively interfere with the other. Consequently, while increasing the injection phase, the net losses of the two supermodes oscillate nearly oppositely, with a slight shift induced by the term ±2ngL/c (Fig. 3*C*). Once the loss variation by the self-injection exceeds the initial loss difference, the two supermodes can lase alternatively, with the switching points at which the two decay rates are exactly the same.

**Fig. 4.**
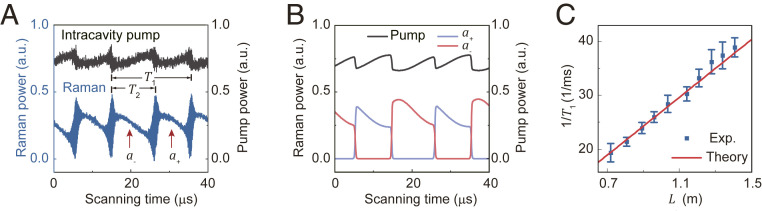
Switchable supermode laser with self-injection. (*A*) Measured intracavity pump power (gray) and Raman laser output (blue) versus scanning time with self-injection. $T_{1}\;∼\;20.6\;\mu s$ is the hopping period, and $T_{2}\;∼\;11.3\;\mu s$ is the duration of the antisymmetric mode lasing in one period. (*B*) Simulated dynamics of lasing mode switching with self-injection. (*C*) Dependence of hopping frequency on optical length L. The error bars denote standard deviation of 10 measurements.

Experimentally, to regulate the injection phase φ, we dynamically tune the Raman laser wavelength via the Kerr and thermo-optic effects by controlling the pump detuning. The beat notes emerge during the pump scanning in Figs. 3*A* and 4*A*, which is also predicted by the theoretical calculation derived in *Materials and Methods*. In the calculation, by extracting the $a_{+}$ and $a_{-}$ components of the hopping supermode laser, it is found that the beat note arises from the transient interference during the switching between an emerging laser and a decaying laser (Fig. 4*B*). As a result, simultaneous lasing from both supermodes occurs only under nonequilibrium evolution, and stable dual-mode lasing cannot be obtained. Besides the temporal beating generation, the self-injection method also provides a strategy to selectively pump and actively switch between the lasing modes. During scanning, the intracavity pump power exhibits a periodic fluctuation due to the loss modulation (Fig. 4 *A* and *B*), indicating that the pump field is not clamped under the self-injection.

To further prove the established theory of lasing dynamics, we experimentally study the hopping period as a function of the feedback length. The hopping period of the supermode lasers ($T_{1}$ in Fig. 4*A*) reads $T_{1}=2\pi /\dot{\varphi }=c\pi /\left(nL\dot{\omega }\right)$, where $\dot{\omega }$ is the frequency shift speed of the Raman laser. Experimentally, the fiber length L between the coupling point and the reflector is changed by cutting the fiber sequentially, and the measured hopping period exhibits a linear dependence on L, consistent with the theoretical result. In each period, the occupation time $T_{2}$ of the antisymmetric mode lasing is longer than that of the other mode, also predicted by the theory (Fig. 4*B*).

**Fig. 5.**
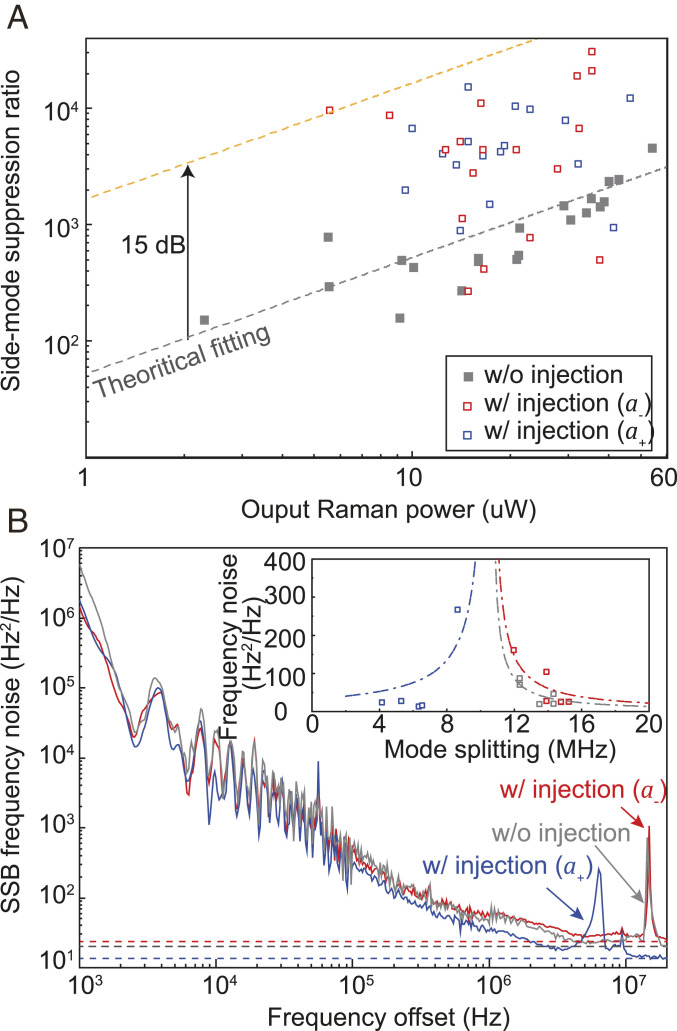
Characterization of the supermode Raman lasers with injection. (*A*) Measurement of side-mode suppression ratio. The gray dashed line indicates theoretical fitting of SMSR in the case without self-injection. (*B*) Spectral density of single-sideband (SSB) frequency noise at different lasing states with and without injection feedback. Values of corresponding white frequency noise are marked with dashed lines. *Inset* shows values of the white frequency noise versus the mode splitting δf. The dashed-dotted line indicates fitting results assuming inverse linear power dependence.

The SMSR of the supermode laser is characterized depending on the self-injection condition. In absence of the self-injection, the SMSR is proportional to the output Raman power (Fig. 5*A*), as predicted by the Langevin analysis (*Materials and Methods*). With the injection feedback, it deviates from the linear power dependence due to the loss difference modulation along the variation of the laser power. Particularly, the SMSR increases 15 dB at a certain laser power, corresponding to an increase of the loss difference of 7.5 dB.

The linewidths of the supermode lasers are measured by a Mach–Zender interferometer with a free spectral range of 5.591 MHz (57). As shown in Fig. 5*B*, the frequency noise reaches a similar level of tens of ${Hz}^{2}$/Hz with and without self-injection, demonstrating that the laser linewidth is narrower than 100 Hz. Besides, the peaks with respect to the Kerr-shifted mode splitting are observed on the noise spectrum. Considering the relationship between the Kerr-modulated mode splitting and the laser power (*Materials and Methods*), the dependence of the measured laser linewidth on the mode splitting δf (Fig. 5 *B*, *Inset*) demonstrates that the linewidth will be broadened as the laser power declines (58).

In summary, we have clarified the controversy between the single-mode nature of stimulated scattering lasers and the previous observed “dual-mode” beat note in near-degenerate supermodes. Experimentally, the pump field is clamped to the mode with lower loss, while the laser is single mode with a SMSR up to 37 dB. The beating phenomenon is retrieved by introducing a self-injection feedback to the microcavity and identified as the transient interference when the lasing mode switches between the supermodes. In this regard, the elusive phenomenon of the temporal beat notes in previous works (8, 9) can be well understood as the interference induced by the existence of a slight reflection in the fiber loops. This work provides an insightful guidance for microlaser-based precision measurements (11) and paves the way to reconfigurable light sources (2) and low-power-consumption optical memories (59).

## Materials and Methods

### Experimental Details.

The transmission spectrum of the supermode for generating the Raman laser is characterized with the following protocol. First, the pump laser is tuned into the mode resonance to excite the Raman laser. Second, a weak probe laser is injected (∼10 μW) into the cavity, whose frequency is in the vicinity of the generated Raman laser. When the scanning range of the probe laser covers the lasing mode, an evident beat signal between the Raman laser and the probe laser is observed. Third, the pump laser is gradually tuned away from the resonance, while the Raman lasing mode is tracked. With the pump laser fully tuned out of resonance, transmission of the Raman lasing mode is recorded, as shown in Fig. 2*B*.

The pump clamping effect in Fig. 2*D* is observed by including a drop coupler, so that the intracavity pump and Raman laser power are measured without the influence of the direct-transmitted pump light. The Raman laser is extracted by a 1,500-nm long-pass filter (Thorlabs FEL1500), while the intracavity pump power is calculated by subtracting the Raman intensity.

The frequency spectrum presented in Fig. 2*E* is measured with a probe laser operating at a nearby higher frequency of the Raman laser. The combined signal is detected by a photodetector and analyzed by an electric spectrum analyzer.

### Influence of Imbalanced Raman Gain in Different Directions.

The Raman gains of the two supermodes are almost the same due to their nearby resonance frequencies, while a tiny gain deviation could result from different gain factors in the counterpropagating Raman waves. In general, the backward Raman gain is slightly lower than the forward Raman gain (2), as a result of the phonon dispersion relation or the self-focusing effect (43, 44). Considering this effect, the eigenvalues of the supermodes are derived,

$$\xi_{\pm }=\omega +i\frac{\kappa }{2}-i|a_{p}{|}^{2}\left(\frac{\delta }{2}+g_{R}\right)\pm \frac{1}{2}\sqrt{4g^{2}+i4g\gamma -\gamma^{2}-|a_{p}{|}^{4}\delta^{2}},$$ [3]

where $g_{R}$ is the Raman gain factor in the backward direction, $|a_{p}{|}^{2}$ is the pump power, and δ is the difference in the Raman gain factor between the two directions. Other parameters have already been defined in the main text. Under the assumption that the gain difference is small (δ≪g), the decay rates of the supermodes are evaluated,

$$\kappa_{\pm }=\kappa \pm \gamma -\frac{|a_{p}{|}^{2}\left(\delta +2g_{R}\right)\pm \left(|a_{p}{|}^{4}\delta^{2}\gamma \right)}{4\left(\gamma^{2}+4g^{2}\right)}.$$ [4]

It is found that the gain difference δ effectively enlarges the intrinsic decay rate difference between the supermodes. Consequently, the gain difference can be treated as an additional slight decay rate difference and will not affect the conclusion of the Raman lasing spectrum in the main text.

### Dissipation of the Supermodes with Self-Injection.

Through the evolution equation −idΦ/dt=HΦ under the traveling-wave basis $\Phi ={\left(a_{cw},a_{ccw}\right)}^{T}$, the system Hamiltonian is obtained as shown in Eq. **1**, where the self-injection term reads $i\tilde{r}\kappa_{in}$. Without self-injection, eigenfrequencies of a microcavity are ω±g. By introducing self-injection, considering that $\left|g|\gg |\gamma /2|,\;|\tilde{r}\kappa_{in}\right|$, the frequency of the reinjected wave can be approximated as ω±g, depending on the wave from which supermode. As a result, the complex reflectivity $\tilde{r}=e^{2in\left(\omega \pm g\right)L/c}$. For the symmetric mode whose eigenfrequency is ω+g, based on Eq. **1**, the eigenvalues of the system with self-injection can be derived by preserving small quantities of first order, $\xi_{+}=\omega +g+\frac{i}{2}\left(\kappa +\gamma -|\tilde{r}|\kappa_{in}e^{2in\left(\omega +g\right)L/c}\right)$. The other eigenvalue $\xi_{+}=\omega -g+\frac{i}{2}\left(\kappa -\gamma +|\tilde{r}|\kappa_{in}e^{2in\left(\omega +g\right)L/c}\right)$ is rejected because its real part, i.e., frequency, is different from the symmetric mode. In the same way, for the antisymmetric mode, $\xi_{-}=\omega -g+\frac{i}{2}\left(\kappa -\gamma +|\tilde{r}|\kappa_{in}e^{2in\left(\omega -g\right)L/c}\right)$. The mode losses of the two supermodes are $\kappa_{\pm }=\kappa \pm \gamma \mp |\tilde{r}|\kappa_{in}\cos\left(2n\left(\omega \pm g\right)L/c\right)$, corresponding to the imaginary parts of the eigenvalues.

### Kerr Frequency Shift and Beating between the Supermodes.

As revealed in previous literature (2, 22), Kerr nonlinearity shifts frequency of the mode resonance regarding intracavity power. Under the $a_{cw}-a_{ccw}$ basis, the following Hamiltonian for the Kerr effect is added,

$$H_{Kerr}=\left(\begin{array}{cc} -2M\left(P_{+}+P_{-}\right) & -M\left(P_{+}-P_{-}\right)\\ -M\left(P_{+}-P_{-}\right) & -2M\left(P_{+}+P_{-}\right) \end{array}\right),$$ [5]

where M is the Kerr nonlinear coefficient, $P_{+}=|a_{+}{|}^{2}$ and $P_{-}=|a_{-}{|}^{2}$. With a weak reflection $r\kappa_{in}\ll \kappa$, the frequency difference between the eigenmodes $a_{+}$ and $a_{-}$ denotes

$$\delta f_{\pm }=2g-2M\left(P_{+}-P_{-}\right).$$ [6]

It is noted that in a steady state, either $P_{+}$ or $P_{-}$ does not vanish. With g>0 and M>0, the beating frequency of the antisymmetric mode is larger than that of the symmetric mode. Also, the equation directly links the changing of δf and Raman laser power.

As can be seen in Fig. 2*E*, when the antisymmetric mode is stimulated, the mode splitting $\delta f_{-}=12.5$ MHz is larger than the passive mode splitting. During the lasing switching, the laser output manifests itself as a spike-like envelope (shown in Fig 3*A*), and the emission intensities in the two supermodes vary alternatively. Therefore, the two supermodes experience different shifts due to the Kerr effect, resulting in the beating frequency continuously switching from $\delta f_{-}$ to $\delta f_{+}$ or vice versa, according to the switching of the lasing modes. The beating frequency in Fig. 3*B* slightly deviates from the passive mode splitting, because of the different power changing in the two supermodes.

### SMSR of the Supermode Raman Laser.

To quantify SMSR in the supermode laser, under the $a_{+}-a_{-}$ basis, a Langevin term (60) is added to the coupled-mode equations,

$$\frac{d}{dt}a_{\pm }=\left(-\frac{\kappa_{\pm }}{2}+i\Omega_{\pm }+G|a_{p}{|}^{2}\right)a_{\pm }+F_{\pm }\left(t\right),$$ [7]

where $\kappa_{\pm }$ is the dissipation rate of the supermodes as derived in the main text, $\Omega_{\pm }$ is the resonant frequency, and F(t) is a stochastic Langevin force with $\left\langle F_{\pm }\left(t\right)F_{\pm }\left(t^{\prime }\right)*\right\rangle =\kappa_{\pm 0}N_{sp}\delta \left(t-t^{\prime }\right)$, where $N_{sp}$ is a spontaneous emission factor and δ(t) is the Dirac δ function.

To evaluate the power of amplified spontaneous emission (ASE) of the mode below threshold, without loss of generality, we assume that the lasing mode is mode $a_{+}$ and $|a_{-}{|}^{2}\approx 0$. With the gain in clamping condition $\kappa_{+}/2=G|a_{p}{|}^{2}$, by applying the Fourier transform, the resulting power spectral density reads

$$\left\langle {ã}_{-}\left(\omega \right){ã}_{-}{\left(\omega \right)}^{*}\right\rangle =\frac{\kappa_{-0}N_{sp}}{{\left(\omega -\Omega_{-}\right)}^{2}+\frac{|\kappa_{+}-\kappa_{-}{|}^{2}}{4}}.$$ [8]

As SMSR is defined as the ratio of the total power of the central peak with the peak power of the nearest mode (61), the calculated SMSR reads

$$R_{\pm }=\frac{|\kappa_{+}-\kappa_{-}{|}^{2}|a_{\pm }{|}^{2}}{8\pi \kappa_{\mp 0}N_{sp}f_{BW}},$$ [9]

where $f_{BW}$ is the resolution bandwidth of the spectral analyzer. In the experiment, a resolution bandwidth of 100 Hz is used in Fig. 5.

### Numerical Calculations of the Supermode Lasing Dynamics.

With the slowly varying field amplitude $c_{p}=a_{p}e^{-i\omega_{p}t}$, $c_{\pm }=a_{\pm }e^{-i\omega_{\pm }t}$, dynamics of the Raman lasing in supermodes are (39)

$$\frac{d}{dt}c_{p}=\left[-\frac{\kappa_{p}}{2}+i\delta \omega -G\left(|c_{+}{|}^{2}+|c_{-}{|}^{2}\right)\right]c_{p}+f_{in},$$ [10]

$$\frac{d}{dt}c_{\pm }=\left(-\frac{\kappa_{\pm }}{2}+G|c_{p}{|}^{2}\right)c_{\pm },$$ [11]

where $|c_{p}{|}^{2}$, $|c_{\pm }{|}^{2}$ are photon number of the pump mode and the pair of supermodes, respectively. $\kappa_{p}$ is dissipation rate of the pump mode. δω is laser-cavity detuning. G is Raman gain coefficient, and $f_{in}$ is pump input. $t=2nL/c_{0}$ is the additional traveling time of the reinjected wave and $\omega_{\pm }=\left(\delta_{t}\pm g\right)$ is the frequency of supermodes, respectively.

A simplified thermal-diffusion equation is utilized to correct the laser-cavity detuning δω by $\delta \omega =\delta_{p}-\delta_{t}$, where $\delta_{p}$ denotes cold cavity detuning and $\delta_{t}$ denotes thermal frequency shift,

$$\begin{array}{cc} \frac{d}{dt}\delta_{t}= & -\kappa_{t}\delta_{t}+2\omega_{p}\frac{n_{T}}{n_{0}}\frac{G\hbar \left(\omega_{p}-\omega_{r}\right)}{C}|c_{p}{|}^{2}\left(|c_{+}{|}^{2}+|c_{-}{|}^{2}\right)\\ & +2\eta \omega_{p}\frac{n_{T}}{n_{0}}\frac{\hbar \omega_{p}}{C}|c_{p}{|}^{2}+2\eta \omega_{p}\frac{n_{T}}{n_{0}}\frac{\hbar \omega_{r}}{C}\left(|c_{+}{|}^{2}+|c_{-}{|}^{2}\right). \end{array}$$ [12]

Here, $\kappa_{t}$ is the thermal diffusion rate, the second heating term is a result of inelastic phonon scattering (62), and the third term denotes linear absorption. η is the fraction of the energy transformed into the heat. Finite-element simulations give a heat capacity of C=0.449 nJ/K and $\kappa_{t}/2\pi =330$ Hz.

The parameters used in the theoretical model and the numerical simulation are given below. In Fig. 3, the coupling strength g/2π=5.49 MHz, the unperturbed mode loss κ=3.99 MHz, the additional decay rate induced by side scattering γ=0.06 MHz, the reflectance $r^{2}=0.033$, the coupling loss $\kappa_{in}/2\pi =2$ MHz, and the output fiber length L=1.03 m with the refractive index n=1.45. In Fig. 4, apart from the coupling loss $\kappa_{in}/2\pi =5.5$ MHz and the output fiber length L=1.22 m, the other parameters are the same. In addition, the lasing threshold of 225 μW gives G/2π=0.015 Hz. Other experimental parameters are pump frequency $\omega_{p}/2\pi =203$ THz, Raman frequency $\omega_{r}/2\pi =190$ THz, pump power 1 mW. η is estimated as 1/500. Constants are thermo-optic coefficient $n_{T}=1.2\times 10^{-5}$/K and refractive index $n_{0}=1.45$.

## Data Availability

All study data are included in this article.
